# Supplementary material for: Development of a Novel Method for the Clinical Visualization and Rapid Identification of Multidrug-Resistant Candida auris
Source: Microbiol Spectr. 2023 Apr 26;11(3):e04912-22. doi: 10.1128/spectrum.04912-22 (PMC10269898; doi:10.1128/spectrum.04912-22)
Supplement: Supplemental file 1 — Supplemental material. Download spectrum.04912-22-s0001.pdf, PDF file, 1.2 MB [file spectrum.04912-22-s0001.pdf]

Supplemental Materials

Table S1. List of experimental strains used in this study

Name	Strain number	Source	
C. auris	CBS10913	Clade II	
C. auris	CBS12766		
C. auris	CBS12767		
C. auris	CBS12768		
C. auris	CBS12769		Centraalbureau voor Schimmelcultures
C. auris	CBS12770	Clade I	
C. auris	CBS12771		
C. auris	CBS12772		
C. auris	CBS12773		
C. auris	CBS12774		
C. auris	DSM105988	Clade III	Deutsche Sammlung von Mikroorganismen und Zellkulturen
C. auris	DSM105990	Clade IV	
C. haemulonii	CBS12436		
C. haemulonii	CBS12437		
C. haemulonii	CBS12438		
C. haemulonii	CBS12439		
C. pseudohaemulonii	CBS10004		
C. pseudohaemulonii	CBS12370		
C. pseudohaemulonii	CBS12371	Centraalbureau voor Schimmelcultures	
C. duobushaemulonis	CBS6915		
C. duobushaemulonis	CBS7089		
C. duobushaemulonis	CBS7798		
C. duobushaemulonis	CBS7799		
C. rugosa	ATCC10571		
C. albicans	ATCC10231		
C. neoformans	ATCC32609		
C. parapsilosis	ATCC22019		
C. glabrata	ATCC2001	American Type Culture Collection	
C. guilliermondii	ATCC6260		
E. coli	ATCC47076		
S. aureus	ATCC51811		
E. faecalis	ATCC29212		
C. tropicalis	CICC1253	China Center of Industrial Culture Collection	
C. krusei	CICC1674		
C. dubliniensis	CMCC50042	National Center for Medical Culture Collections	
K. pneumoniae	BNCC330357	BeNa Culture Collection	

Table S2. Primers and probes tested in this study

Name		Sequence (5′-3′)
ITS-1	F1	TTTGAGCGTGATGTCTTCTCACCAATCTTC
	R1	CTACCTGATTTGAGGCGACAACAAAACGAA
ITS-2	F2	CCTGTTTGAGCGTGATGTCTTCTCACCAAT
	R2	TAAGTTCAGCGGGTAGTCCTACCTGATTTG
ITS-3	F3	CATGCCTGTTTGAGCGTGATGTCTTCTCAC
	R3	AGTTCAGCGGGTAGTCCTACCTGATTTGAG
ITS-4	F4	TGCCTGTTTGAGCGTGATGTCTTCTCACCA
	R4	TAAGTTCAGCGGGTAGTCCTACCTGATTTG
ITS-5	F5	ACCAATCTTCGCGGTGGCGTTGCATTCACA
	R5	AGTTCAGCGGGTAGTCCTACCTGATTTGAG
ITS-6	F6	GATCATTATTGATATTTTGCATACACACTG
	R6	AGATCCGTTGTTGAAAGTTTTCTTTATAGT
ITS-7	F7	TATTGATATTTTGCATACACACTGATTTGG
	R7	ACCAAGAGATCCGTTGTTGAAAGTTTTCTT
ITS-8	F8	GATATTTTGCATACACACTGATTTGGATTT
	R8	CAATGTGCGTTCAAAGATTCGATGATTCAC
ITS-9	F9	CTATAAAGAAAACTTTCAACAACGGATCTC
	R9	TTCGATGATTCACGTCTGCAAGTCATACTA
ITS-10	F10	ACTGATTTGGATTTTAAAACTAACCCAACG
	R10	TTCAAAGATTCGATGATTCACGTCTGCAAG
	P	FAM—ACTGATTTGGATTTTAAAACTAACCCAACG[THF]TAAGTTCAACTAAAC—C3spacer
ITS-10′	F10′-1	AAGGATCATTATTGATATTTTGCATACACA
	F10′-2	TCATTATTGATATTTTGCATACACACTGAT
	F10′-3	ATTGATATTTTGCATACACACTGATTTGGA
	F10′-4	TATTTTGCATACACACTGATTTGGATTTTA
	R10′	Biotin—TTCAAAGATTCGATGATTCACGTCTGCAAG

FAM/Biotin is a fluorophore; THF is tetrahydrofuran; C3-spacer is the blocking group

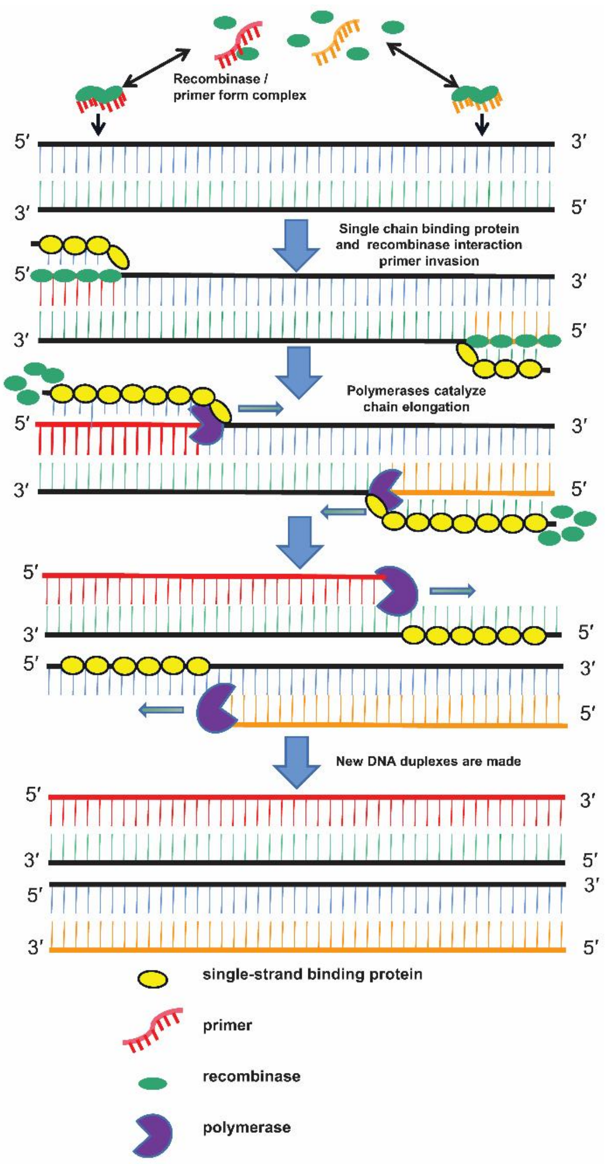

Fig. S1. Schematic diagram of the RAA reaction.

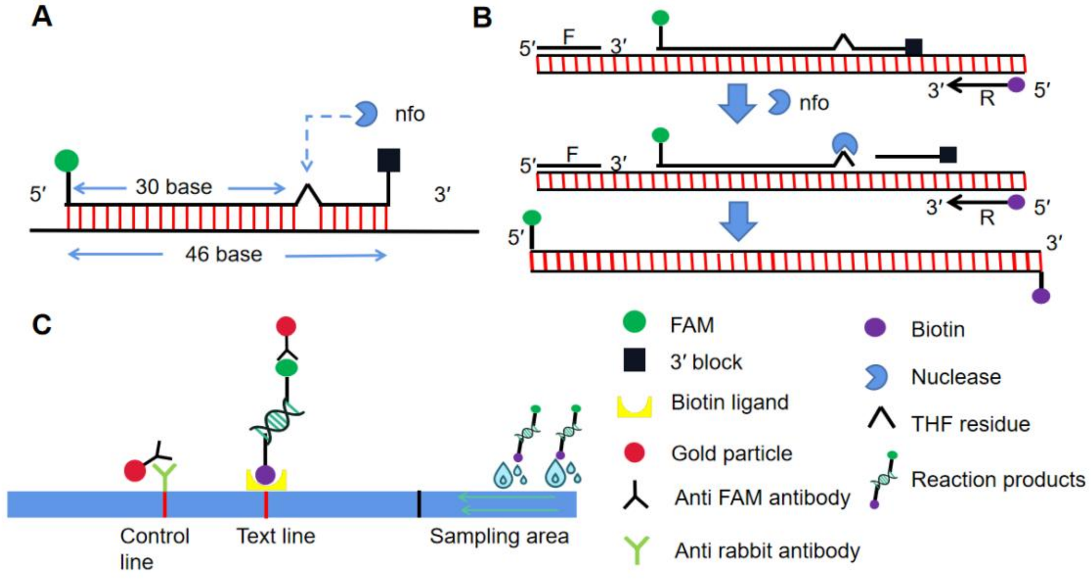

Fig. S2. Schematic diagram of RAA-LFS. (A) RAA-NFO probe design. The 5′-end of the probe is labeled with an antigenic marker, usually an FAM group or hydroxyl fluorescein. Nucleotides containing substitutions of base nucleotide analogs inside (such as THF- sometimes called 'dSpacer') are represented. The polymerase extension blocking group at the 3′-end (such as C3-spacer, phosphate group, or dideoxynucleotide) is illustrated. **(B)** The fluorescently labeled probe binds to the template. The probe is then enzymatically digested by the endonuclease. It is amplified together with biotin-labeled primers to form a fluorescently labeled fragment with biotin labels at both ends. **(C)** Schematic representation of the lateral flow strip (LFS) workflow. The diluted product is added dropwise to the sample pad. Both ends of the amplification product are labeled with biotin and FAM, respectively. FAM binds to AuNPs, and biotin binds to streptavidin when passing through the detection line of streptavidin, whereas the other end passes through AuNPs, giving a positive signal.

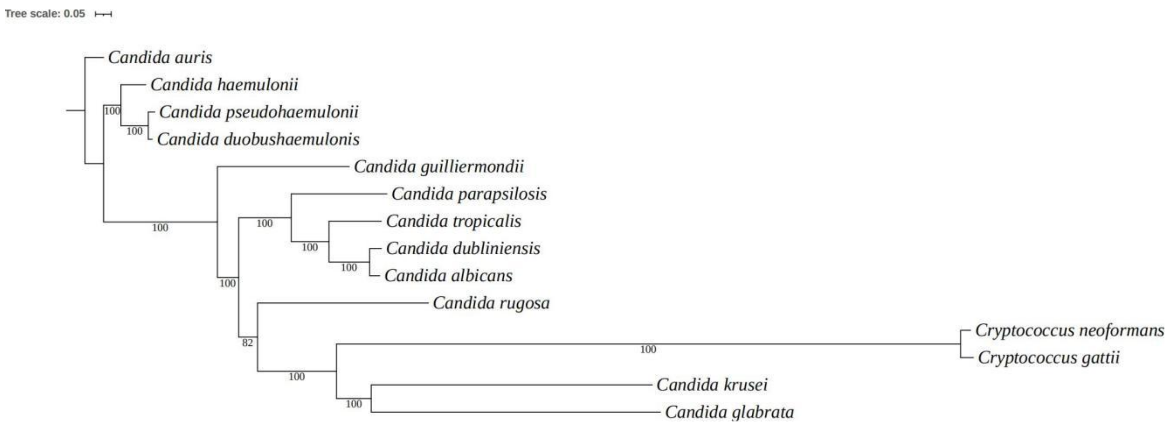

Fig. S3. Whole-genome phylogeny of *Candida auris* and other invasive *Candida* species. OrthoFinder was used to identify and cluster orthologous genes. A maximum-likelihood phylogenetic tree was constructed using iqtree2. The phylogeny was rooted in *C. auris*. The numbers in the tree structure indicate nodes with ultrafast bootstrap values.

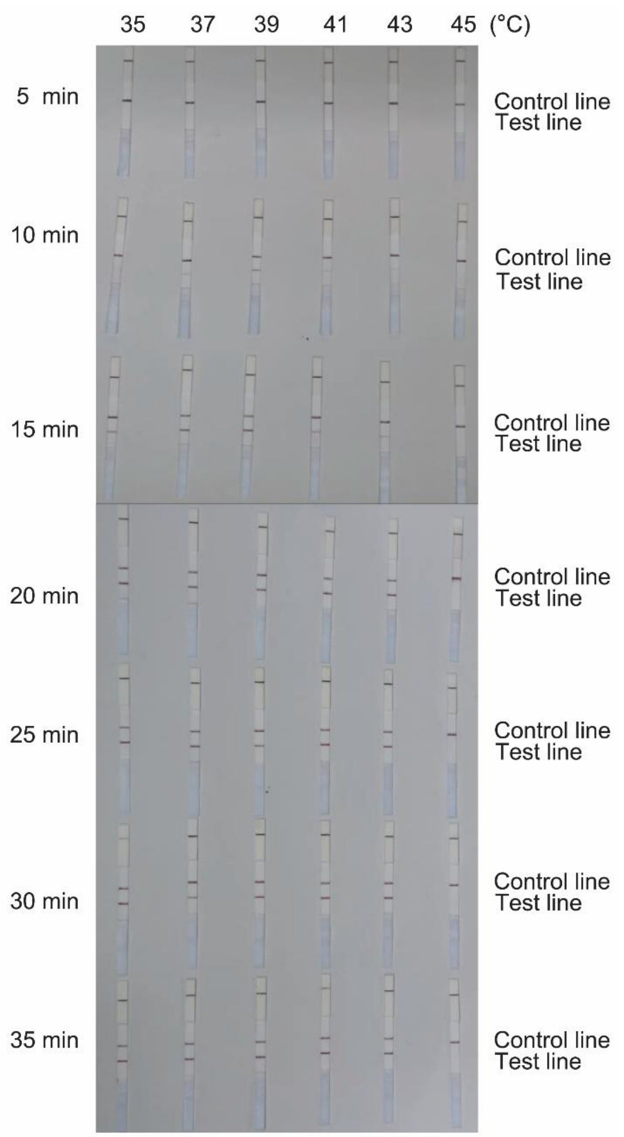

Fig. S4. Optimal reaction temperature and time of the RAA-LFS system. (A) RAA-LFS test results under different reaction temperatures and incubation periods. The temperatures under which the RAA reactions were performed are indicated at the top of each strip. The incubation period of each RAA reaction is indicated on the left side of each row. *Candida auris* genomic DNA was used as the amplification template. The positions of the control and test lines are indicated on the right side.

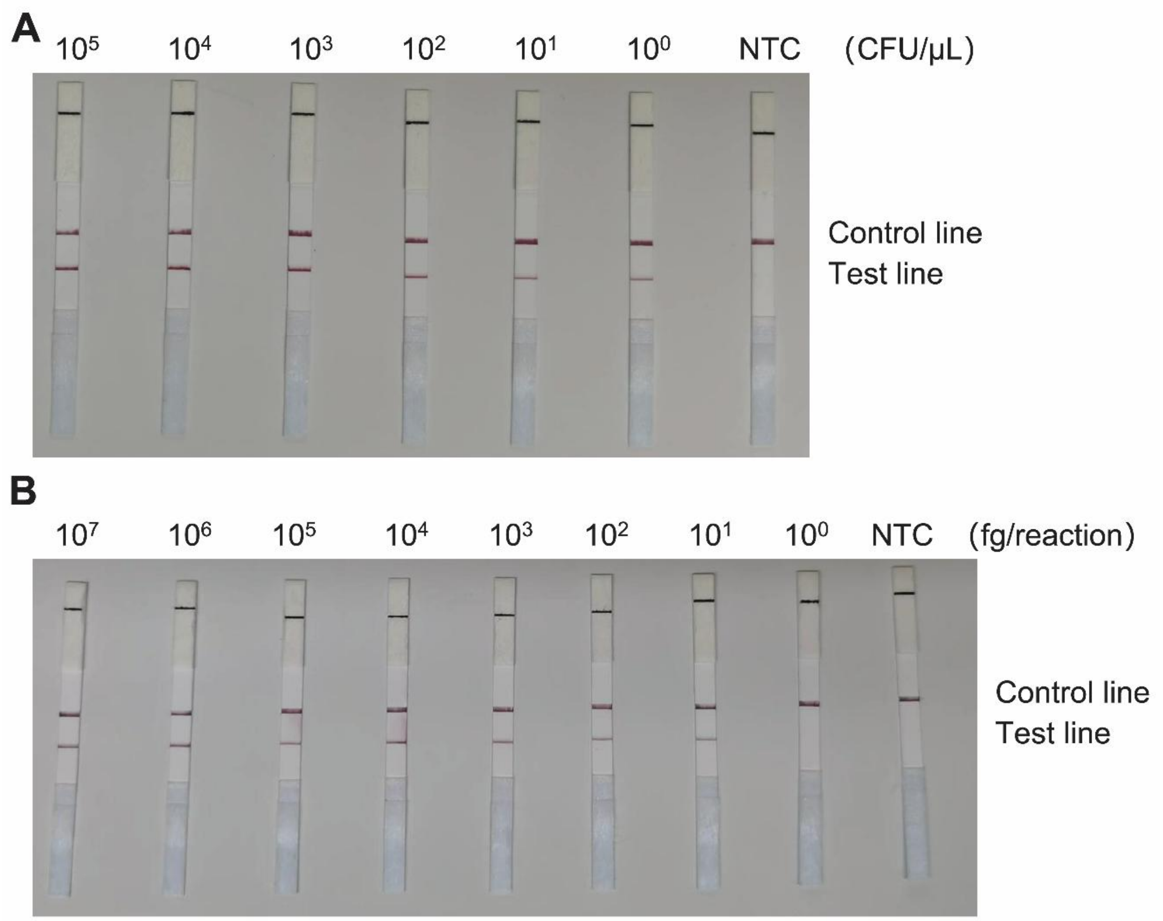

Fig. S5. Detection limit of the RAA-LFS system for *Candida auris*. (A) LFS results of RAA amplifications using different amounts of *C. auris* culture. The amounts (in CFU) added to the RAA reactions are indicated at the top of each strip. **(B)** LFS results of RAA amplifications using different amounts of *C. auris* genomic DNA. The amounts added to the RAA reactions are indicated at the top of each strip. NTC, no-template control. All reactions were performed at 37 °C for 15 min. The positions of the control and test lines are indicated on the right of the images.

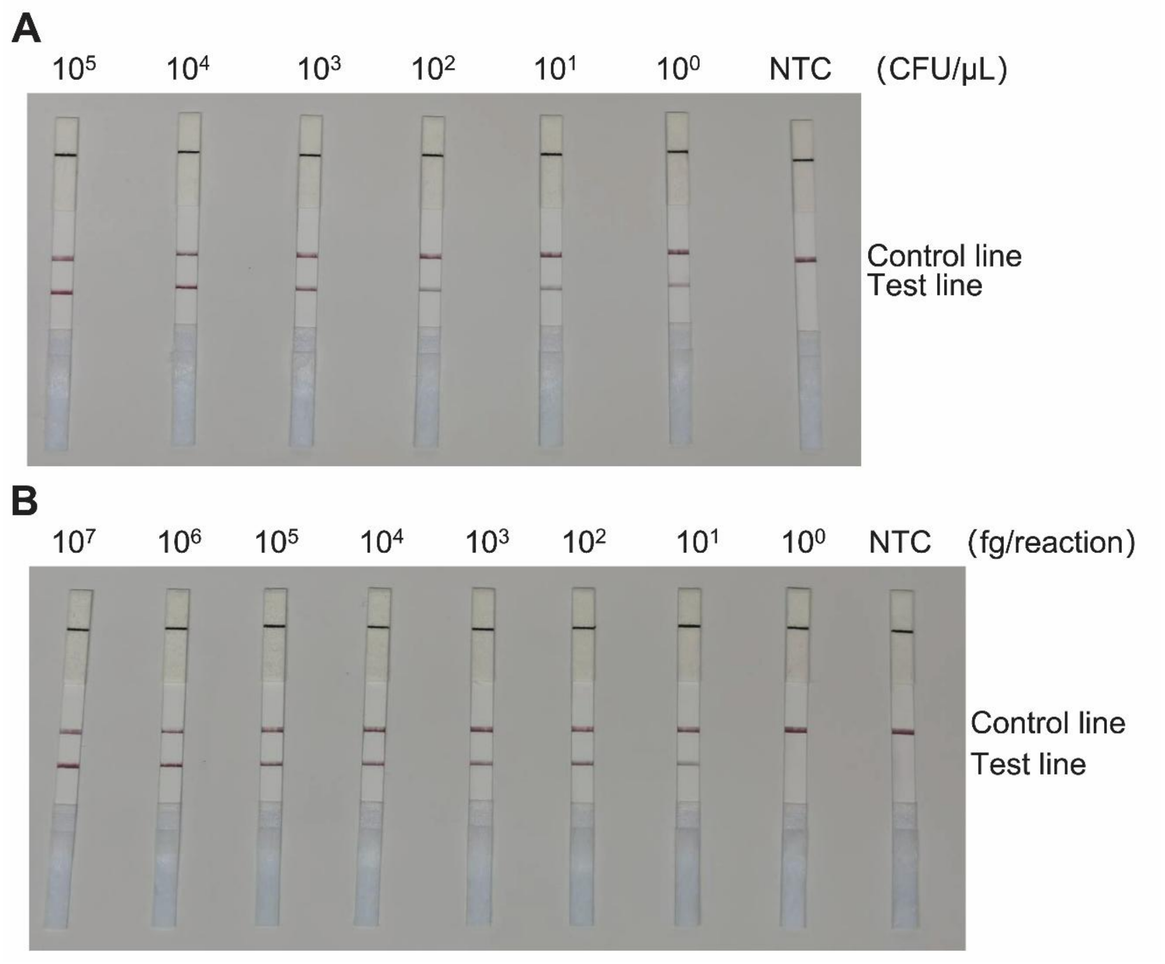

Fig. S6. Detection limit of the RAA-LFS system for *Candida auris* in the presence of other fungi. **(A)** LFS results of RAA amplification of different cultures of *C. auris* after the addition of 10^5 CFU/µL *C. albicans*. The amounts (in CFU) added to the RAA reactions are indicated at the top of each strip. **(B)** *C. albicans* genomic DNA (1 ng) was added to the reactions in addition to the *C. auris* genomic DNA. The amounts added to the RAA reactions are indicated at the top of each strip. NTC, no-template control. All reactions were performed at 37 °C for 15 min. The positions of the control and test lines are indicated on the right of the images.

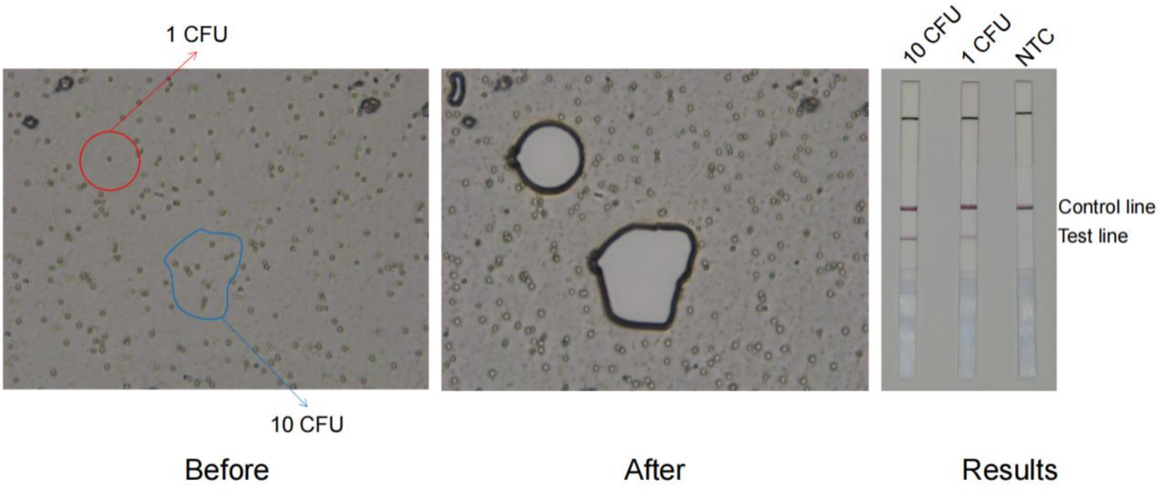

Fig. S7. Results of the RAA-LFS detection system on a single *Candida auris* cell. RAA-LFS detection was performed by cutting single and 10 cells of *Candida auris* by laser microdissection, and the sensitivity of the RAA-LFS detection system was proved to be 1 CFU. The amounts added to the RAA reactions are indicated at the top of each strip. NTC, no-template control. All reactions were performed at 37 °C for 15 min. The positions of the control and test lines are indicated on the right.

A

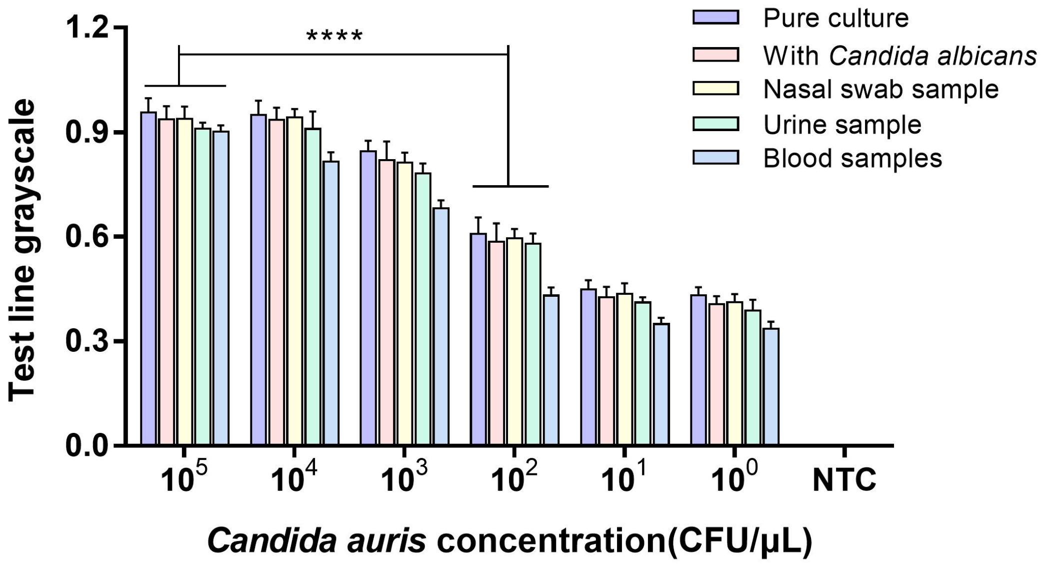

B

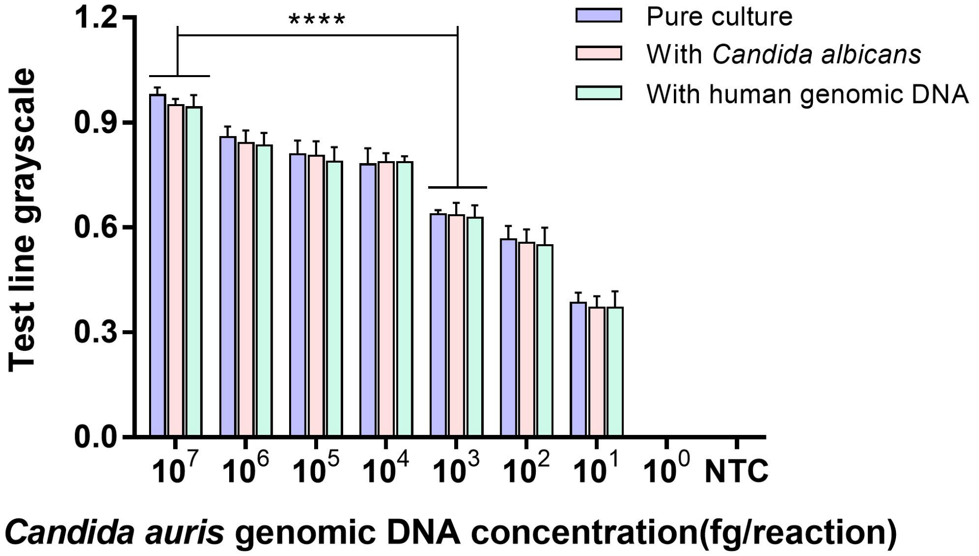

Fig. S8. Different experimental groups of lateral flow strips tested using line-band grayscale analysis. (A) Different groups used different amounts of *Candida auris* culture for the RAA amplified LFS test-line grayscale. **(B)** Test line grayscale of RAA-amplified LFS using different amounts of *Candida auris* genomic DNA in different groups. All experiments were performed in triplicates. Error bars represent standard deviation. Indicated p-values were calculated by the Student's *t*-test (unpaired). ****: $p \leq 0.0001$.

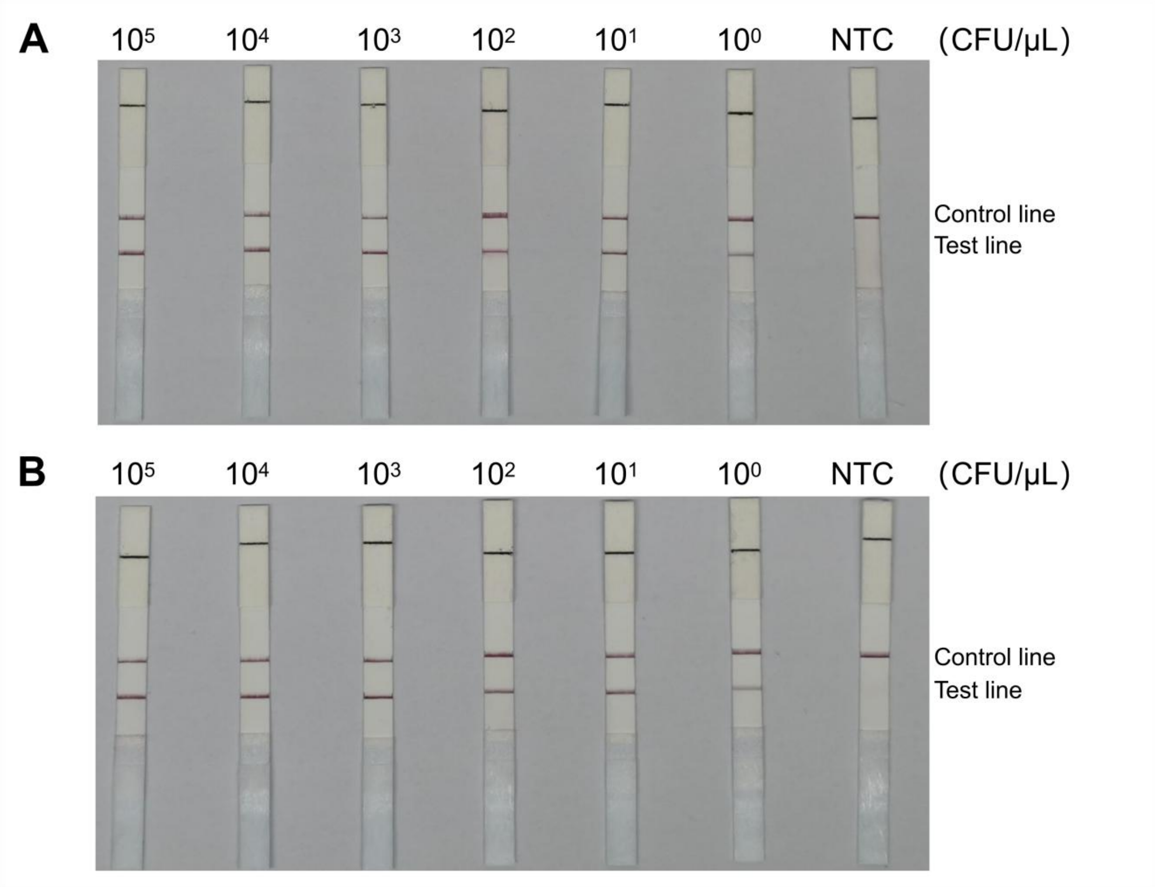

Fig. S9. Testing the RAA-LFS system using nasal swab and urine samples. (A) Detection limits of the RAA-LFS system for *Candida auris* mixed into nasal swab samples. **(B)** Detection limits of the RAA-LFS system for *Candida auris* mixed into urine samples. NTC, no-template control. The positions of the control and test lines are indicated on the right. All reactions were performed at 37 °C for 15 min.

Table S3. Two methods used to test the results of simulated blood samples

Sample Number	qPCR		RAA-LFS
	Ct	+/-	+/-
1	27.03	+	+
2	30.05	+	+
3	N/A	-	-
4	30.98	+	+
5	N/A	-	-
6	32.29	+	+
7	N/A	-	-
8	N/A	-	-
9	33.82	+	+
10	34.94	+	+
11	29.52	+	+
12	N/A	-	-
13	N/A	-	-
14	30.28	+	+
15	31.45	+	+
16	N/A	-	-
17	32.44	+	+
18	33.5	+	+
19	33.93	+	+
20	37.54	+	-
21	N/A	-	-
22	N/A	-	-
23	N/A	-	-
24	26.59	+	+
25	N/A	-	-
26	N/A	-	-
27	N/A	-	-
28	N/A	-	-
29	N/A	-	-
30	24.77	+	+
31	32.36	+	+
32	N/A	-	-

Sample Number	qPCR		RAA-LFS
	Ct	+/-	+/-
33	N/A	-	-
34	34.11	+	+
35	32.33	+	+
36	N/A	-	-
37	N/A	-	-
38	N/A	-	-
39	N/A	-	-
40	N/A	-	-
41	33.82	+	+
42	N/A	-	-
43	N/A	-	-
44	N/A	-	-
45	N/A	-	-
46	N/A	-	-
47	N/A	-	-
48	N/A	-	-

"+" indicates a positive result; "-" indicates a negative result. "N/A," not applicable

Table S4. Two methods used to test the results of the simulated nasal-swab samples

Sample number	qPCR		RAA-LFS
	Ct	+/-	+/-
1	27.96	+	+
2	31.97	+	+
3	N/A	-	-
4	N/A	-	-
5	N/A	-	-
6	N/A	-	-
7	33.33	+	+
8	33.13	+	+
9	32.42	+	+
10	N/A	-	-
11	N/A	-	-
12	N/A	-	-
13	N/A	-	-
14	34.62	+	+
15	32.55	+	+
16	34.58	+	+
17	N/A	-	-
18	34.02	+	+
19	N/A	-	-
20	N/A	-	-
21	N/A	-	-
22	N/A	-	-
23	29.65	+	+
24	N/A	-	-
25	31.14	+	+
26	31.32	+	+
27	N/A	-	-
28	N/A	-	-
29	N/A	-	-
30	N/A	-	-
31	N/A	-	-
32	33.22	+	+

Sample number	qPCR		RAA-LFS
	Ct	+/-	+/-
33	N/A	-	-
34	N/A	-	-
35	N/A	-	-
36	N/A	-	-
37	34.69	+	+
38	32.28	+	+
39	33.57	+	+
40	35.13	+	+
41	N/A	-	-
42	31.09	+	+
43	N/A	-	-
44	N/A	-	-
45	N/A	-	-
46	29.65	+	+
47	N/A	-	-
48	N/A	-	-

"+" indicates a positive result; "-" indicates a negative result. "N/A," not applicable

Table S5. Two methods used to test the results of simulated urine samples

Sample number	qPCR		RAA-LFS
	Ct	+/-	+/-
1	30.11	+	+
2	N/A	-	-
3	N/A	-	-
4	31.62	+	+
5	31.93	+	+
6	N/A	-	-
7	N/A	-	-
8	N/A	-	-
9	32	+	+
10	32.68	+	+
11	30.02	+	+
12	N/A	-	-
13	N/A	-	-
14	N/A	-	-
15	N/A	-	-
16	N/A	-	-
17	28.63	+	+
18	30.22	+	+
19	N/A	-	-
20	30.5	+	+
21	31.33	+	+
22	N/A	-	-
23	N/A	-	-
24	N/A	-	-
25	N/A	-	-
26	N/A	-	-
27	32.64	+	+
28	29.98	+	+
29	29.95	+	+
30	30.85	+	+
31	31.81	+	+
32	N/A	-	-

Sample number	qPCR		RAA-LFS
	Ct	+/-	+/-
33	N/A	-	-
34	32.79	+	+
35	N/A	-	-
36	N/A	-	-
37	N/A	-	-
38	N/A	-	-
39	N/A	-	-
40	32.5	+	+
41	30.12	+	+
42	31.2	+	+
43	N/A	-	-
44	N/A	-	-
45	N/A	-	-
46	N/A	-	-
47	31.36	+	+
48	31.64	+	+

"+" indicates a positive result; "-" indicates a negative result. "N/A," not applicable
